# Ketone bodies – causes and effects of their increased presence in cows’ body fluids: A review

**DOI:** 10.14202/vetworld.2021.1492-1503

**Published:** 2021-06-11

**Authors:** Piotr Guliński

**Affiliations:** Institute of Animal Production and Fisheries, Siedlce University of Natural Sciences and Humanities, 08-110 Siedlce, ul. Prusa 14, Poland

**Keywords:** dairy cattle, ketone bodies, ketosis, metabolic disorders

## Abstract

Ketosis is the most common metabolic disease in high-performance dairy cows during the first 6-8 weeks of lactation. Its main symptoms include an excessive amount of so-called ketone bodies in a cow’s body fluids. Ketone bodies consist of β-hydroxybutyric acid (βHBA), acetoacetic acid, and acetone. βHBA is the main component with its share of the total volume of ketone bodies in the blood of about 70%. Clinical symptoms of ketosis in cows include loss of appetite, preference for forage to concentrated feed, and acetone odor in their mouth and urine. Those symptoms are accompanied by a production drop, an increase of concurrent illness (*mastitis*, metritis, and displaced abomasum), and poor reproductive performance. One of the ketosis characteristic effects is an increase in the level of fat in milk (>5%), while protein levels decrease (<2.9%). In the case of subclinical ketosis (SCK), the fat–protein ratio in milk is increased to above 1.4:1. The current consensus for SCK is to consider a cutoff point of βHBA to be at least 1.2 mmol/L in blood plasma. Ketosis prevention is based on keeping perinatal cows in good condition, that is, with around 3.5 points in the five-point body condition scoring, carefully balancing food doses during the first 2 months of lactation with the correct energy–protein ratio. Glucose precursor products should be administered orally, in particular to at-risk herds. Ketosis occurs in 7-14% on average of the total number of cows in a herd. In general, data on the prevalence of SCK vary considerably, depending on their source. Moreover, the problem is mostly observed in poorly-fed animals with high milk production potential. The objectives of this review are to reveal the current situation of ketosis prevalence, the possibility of diagnosis, consequences in dairy cows and to provide some recommendations for ketosis treatment and prevention.

## Introduction

Ketosis is the most common metabolic disease in high-performance dairy cows during the first 6-8 weeks of lactation. Its main symptoms include an excessive amount of so-called ketone bodies in a cow’s body fluids. Ketone bodies consist of β-hydroxybutyric acid (βHBA), acetoacetic acid (ACAC), and acetone (AC).

The significance of this study focuses on the problem of ketosis which is the main metabolic disease in dairy herds. Ketosis is a disease with a severe impact on animal performance and consequently on the economic well-being of dairies. According to a study [1] in the typical Dutch context, the actual average herd level costs of clinical and subclinical ketosis (SCK) were €3613 and €7371 per year for default and high-risk farms, respectively.

The main objectives of this review are:

- To reveal the current situation of ketosis prevalence in selected countries
- To describe the impact of the increased presence of ketone bodies in body fluids on cows production
- To describe the consequences of negative energy balance (NEB) in dairy cows; and
- To provide some recommendations for ketosis treatment and prevention.

### Ketone Bodies – the Causes of their Increased Presence in Body Fluids and Ketosis Prevalence

**Figure 1:**
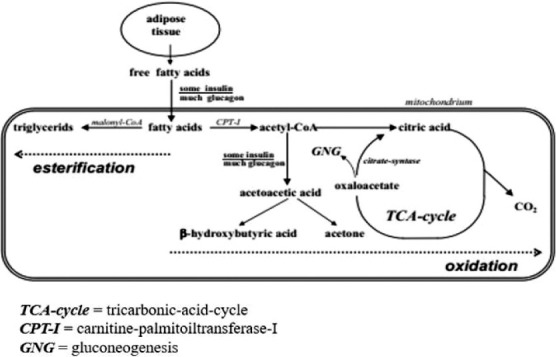
Connection, formation, and transformation of ketone bodies related to TCA-cycle [2].

**Figure 2:**
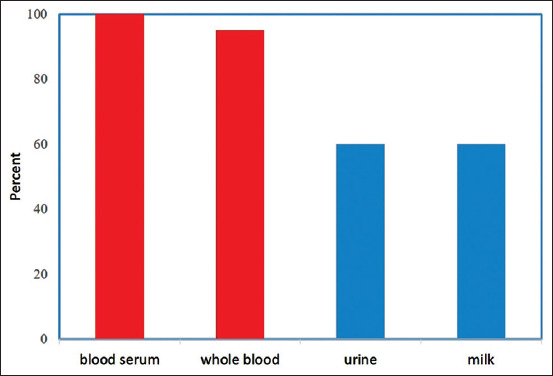
Relative βHBA share in cows body fluids [5].

Ketone bodies are a group of organic chemicals that are intermediary fat metabolites. They are an alternative product of free fatty acid oxidation in the liver, and the formation process is termed *ketogenesis*. They arise when the body derives energy from fat molecules instead of drawing it from glucose. Some of the latter’s molecules can then be converted into ketone bodies (e.g. βHBA, ACAC, and AC; Figure-1) [2] when the liver rapidly metabolizes fatty acids into acetyl-CoA. In the case of ketosis (βHBA concentration in blood, >1200 mmol/L), the concentration of individual ketone compounds in the blood of cows was βHBA, 1719 mmol/L; ACAC, 236 mmol/L; and an AC, 356 mmol/L [3]. Excess of ketone bodies is excreted in urine and milk (Figure-2) [4,5].

Hyperketonemia is a worldwide problem for high-yielding cows. It is the most important metabolic disease in dairy cows in Poland and other countries, being their typical production disease. This is especially true in SCK, a condition in which the animal has elevated levels of ketone bodies in the blood, milk, and urine, with often reduced glucose levels, but does not yet show clinical signs. Cows most often develop ketosis during the first 2 months of lactation. Assessing βHBA is most commonly used for disease diagnosis. Its level, alongside the increased AC and ACAC concentration in the body fluids, is the most characteristic of the ketosis symptoms [5-7].

The gold standard method for ketosis testing in dairy cows is through measuring the levels of blood β-hydroxybutyrate [5,8,9]. Being one of the primary ketones presents in the bloodstream, βHBA is a useful biomarker for ketosis. Moreover, it is more stable in blood than acetone or acetoacetate. The current consensus is to consider a βHBA cutoff point of at least 1.2 mmol/L for SCK [10]. According to Suthar *et al*. [11], SCK is defined as concentrations of βHBA ≥1.2-1.4 mmol/L in blood, and the latter considered the threshold condition for clinical ketosis (CK). Moreover, some studies [9,12] point out that SCK ketone levels between 1.2 and 2.9 mmol/L indicate a possibility of advancing cases of ketosis and health risks during early lactation. Thus, while the critical point for the SCK occurrence is the βHBA level in the blood of >1.2 mmol/L, a critical βHBA level above ca. 3.0 mmol/L in the blood is commonly used for CK. In a study of Wang *et al*. [13], the βHBA level in the plasma cows with CK and SCK was 2.61±0.49 and 1.24±0.34 mmol/L with 0.74±0.67 mmol/L for healthy cows.

The effects of environmental factors on the level of ketone bodies in Holstein cows were studied [14]. They used 264,221 milk samples collected from Korean cows at different lactation stages to determine their composition, including acetone and βHBA content. Changes in the diet were found to affect ketone bodies concentration the most. Differences were also found between the samples collected during morning and evening milking with both βHBA and AC levels increasing in the evening. Ketone body concentrations increased during the first lactation stage and then decreased, only to rise slightly at the end of the milking period. βHBA concentration was affected by the season of the year, but not acetone concentration. In addition, βHBA levels slightly increased overparities. The authors conclude that some environmental factors can be used to control ketone body metabolism, which keeps ketosis cases at a low level.

Data on the SCK prevalence generally vary considerably, depending on their source. The problem is mostly observed in poorly-fed animals with high milk production potential. Among cows assessed for βHBA, a level exceeding 1.4 mmol/L was found in at least 10% of the animals in Poland. However, according to Guliński [4], 20%, 16%, and 23% of cows in the first, second, and third and subsequent lactations, respectively, were affected similarly. The prevalence of subclinical ketoses in bovine herds ranged from 30% to 50% in North America [15].

SCK prevalence and production-related clinical diseases in early lactating cows in various countries across the world, except North America and Western Europe, were investigated by Brunner *et al*. [16]. Twelve countries in South and Central America (Argentina, Brazil, Chile, Colombia, and Mexico), Africa (South Africa), Asia (Thailand and China), Eastern Europe (Russia and Ukraine), as well as Australia and New Zealand, were assessed. Data from 8902 cows from 541 commercial dairy farms were obtained. A minimum of 5 cows per farm was blood sampled and examined once after parturition of up to day 21 of lactation. The SCK prevalence was 24.1% (range, 8.3-40.1%) across all investigated countries. Despite differences in the production systems across countries and variations between individual farms within a region, the authors pointed out that data on SCK prevalence aligned with observations in Western European and North American dairy herds [16]. Data from 1693 Holstein–Friesian primiparous cows in Germany recorded within the first 180 days of lactation by Buttchereit *et al*. [17] indicated that disease frequencies (percentage of cows with at least one case) were 9.7% for metabolic disorders.

A total of 299 Holstein cows were evaluated during early lactation (from calving to day 30 of lactation) in 15 herds in the western region of Santa Catarina state in Brazil [18]. Blood samples were collected for βHBA measurement. The SCK cutoff point in that research was when serum βHBA concentration was >1.2 mmol/L. The results revealed a 9% occurrence of SCK. Although no significant differences were generally observed in SCK prevalence among cows with different production levels, the rate of this disorder was almost double in cows yielding more than 30 L/day than in animals producing 15-30 L/day (14.9% and 6.9%, respectively).

SCK prevalence was greatest in high-yielding dairy cows and those with two or more lactations [19]. Moreover, the relationship between the fat–protein (F/P) ratio was examined, on the one hand, and metabolic disorders and the milk yield, on the other hand [20]. The average daily milk yield and F/P ratio from 908 samples were 35.25 and 1.19 kg, respectively. The prevalence of ketosis was found to be highest at the beginning of the lactation, while acidosis cases decreased over the first milking period (150 days). In the 1^st^ and 5^th^ months of lactation, the proportion of ketosis risk was 22% and 2.78%, respectively. The authors linked the increase of ketosis incidence to an increase in the milk yield in the 1^st^ month of lactation. Increased amounts of ketone bodies in the milk were recorded because cows consumed less energy and dry matter than needed. The authors concluded that a higher F/P ratio was related to both an increased milk yield and risk of SCK.

Urine samples were collected from 479 Polish Holstein–Friesian (PHF) cows [21]. The samples were tested using KRULAB test strips. It was found that 36.2% of them contained ketone bodies that could indicate various forms of ketosis. Moreover, the first lactation Holstein cows were studied and ketosis prevalence was found to be highest in the 1^st^ day after calving with 33% of cows affected [22]. The highest ketosis incidence was 10 days later than for primiparous cows for animals in their second and later lactations. Similar to ketosis, the highest incidence of acidosis was at the beginning of lactation and it decreased later. Moreover, 4838 observations in 10 PHF herds were carried out in Poland and the average F/P ratio of 1.26 was recorded [23]. It was observed that in 3313 milk samples (68.5%), the F/P ratio ranged from 1.1 to 1.4:1, indicating adequate nutrition and daily rations. However, 8.2% of the observations indicated the prevalence of acidosis in the cow population, and 19.9% of the observations suggested the occurrence of SCK and 3.3% of the milk samples were from cows affected by CK. According to Vince *et al*. [24], the SCK prevalence was 15.8% in Holstein cows from the central, northern, northwestern, and eastern parts of Croatia. It was found that multiparous cows had a 1.41 times more chance of being affected by SCK than primiparous cows.

### Consequences of NEB in Dairy Cows – the Importance of Optimal Condition of Cows in the Perinatal Period in Ketosis Prevention

Ketosis is a disease with a severe impact on animal performance and consequently on the economic well-being of dairies. Prevention is usually less costly than treatment because the latter is associated with production losses. An essential element in ketosis prevention is keeping cows in good condition during the perinatal period. The condition of cows during this period should be between 3.0 and 3.5, assessed in the five-point body condition scoring (BCS) system [25]. Overweight cows with BCS of over four points at this time are at high risk for many problems, such as difficult calving, placenta retention, ketosis, and postpartum paralysis. BCS is linked to metabolic changes during the postpartum period, and its elevated value at calving is a major risk factor for ketosis. Cows with elevated BCS at calving (BCS≥ 4.0) have elevated levels of circulating ketone bodies in plasma. They are at the highest risk of developing clinical and SCK compared with cows classified as either a moderate or thin BCS before calving.

Moreover, cows starting lactation in a condition that is too poor, below 2.5 points, do not have energy reserves. The risk of perinatal diseases, in this case, is much lower, but the subsequent production and reproduction of such animals will be at a lower level. The milk production peak of such animals is small and the yield correlated with it throughout the lactation will be much lower. Every extra kilogram of milk produced at the lactation peak means about 200 kg more milk throughout the lactation [14].

It is established that as cows enter NEB after calving, a switch toward the increasing use of fatty acids exists as an energy source to conserve limited glucose supplies. This is promoted by several endocrine signaling pathways: IGF1 and leptin concentrations fall, insulin signaling is blocked, and growth hormone and catecholamine secretion promote lipolysis. The rate of body tissue mobilization is influenced by energy input (dry matter intake [DMI]), energy stores (BCS), and energy output (milk production) in the critical period around calving [26].

The proportion of cows starting lactation in breeding practice in optimal condition, that is, between 3.0 and 3.5 points of BCS, varies depending on the source. The body condition of 644 black and white PHF cows with annual milk yield exceeding 10,000 kg was assessed [27]. The percentage of cows with the condition at the beginning of lactation rated as 3.25-3.75 and 2.5-3.9 was 27.3 and 55.1, respectively. Studies on 270 black and white PHF cows were carried out [28], and the average assessment of their condition in the 1^st^ month of lactation, depending on the month of calving, ranged from 2.81 (December to January) to 3.25 (September to November). According to Sablik *et al*. [29], the distribution of BCS of cows with different daily productivity, different ages, and evaluated in different seasons of the year, was significantly related to the housing system. Evaluated in the summer season, cows with the highest daily milk yield (>35 kg) and younger ones (in the second lactation) had the least favorable distribution of condition scores with the lowest average value compared with the other groups. Cows kept on tether more often were found to receive extreme BCS, that is, no more than 2.0 points or 4.0 and more BCS points, compared with cows from the free-stall system. Attention as also given to the role of the housing system for dairy production [30].

**Figure 3:**
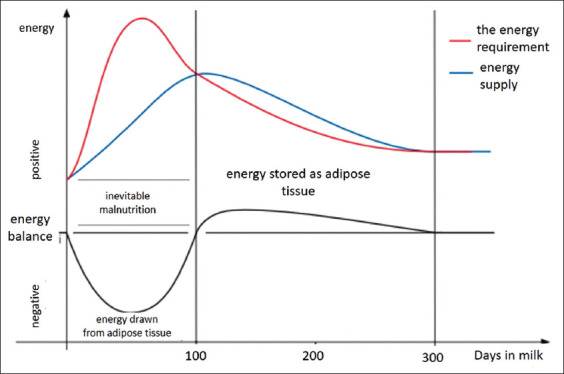
Dairy cows energy balance [14].

The peak of daily milk production is usually 4-6 weeks of lactation, while the peak of DMI is between 9 and 11 weeks of lactation. This situation causes NEB during the first 2 months of lactation (Figure-3). This means that the energy value of feedstuffs is less than its expenditure on milk production. Cows during this period should consume their fat reserves to cover for the energy deficit. Therefore, an adult cow should lose between 0.5 and 1.0 BCS when assessing its condition during the first 2 months of lactation. For 4- to 5-year-old cows with a 3.5-point condition at the time of calving, their weight is reduced during the first 60-80 days of lactation by approximately 0.5-1 kg/day [31]. A loss of 1 kg of body weight (mainly tissue fat) means a loss of 4.92 MCal of energy [32]. Milk with 3.5% of fat contains 0.69 MCal of energy in 1 kg. Thus, 1 kg of fat tissue covers the need for the production of 7.1 kg of milk. The loss of 70 kg of fat tissue in an adult cow is used to produce nearly 500 kg of milk more than the amount of milk produced from the energy contained in feedstuffs [31].

**Figure 4:**
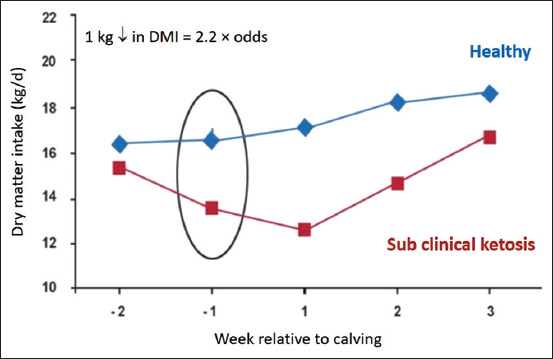
Early detection of endangered cows in the dry phase [33].

Any factor resulting in a reduction of DMI increases the risk of ketosis. Cows with SCK eat less in the week before and 2 weeks after calving (Figure-4) [33]. Around calving, lactating dairy cows naturally decrease DMI due to the advanced gestation stage, as well as metabolic changes which occur in this period. This DMI decrease typically leads to a NEB. During the last week of fetal development, the fetus uses approximately 46% of maternal glucose. The onset of milk production makes this energy shortage even more remarkable. The mammary gland requires a large amount of glucose for milk lactose synthesis when lactation starts.

Glucose blood concentration is considered to be a direct indicator of energy balance in the organism. It has an important role in high-producing dairy cows because certain vitally important cell types and metabolic pathways have to rely on glucose as the only energy substrate. In ruminants, blood glucose regulation differs from the regulation in most monogastric animals. In ruminants, volatile fatty acids from the gastrointestinal tract are the major energy source rather than direct glucose sources. Within the ruminants, high-producing dairy cows occupy a special position regarding glucose metabolism which is related to increases in energy requirements driven by both fetal needs and lactogenesis [34].

Daily glucose requirements for cows are 0.5 kg/day to maintain body functions and about 0.05 kg for each liter of milk. Thus, a cow producing 40 L per day needs 2.5 kg of glucose. Moreover, this amount should be systematically delivered during the day. The main glucose precursors are 70% propionic, lactic, and pyrogenic acids.

### Symptoms and Types of Ketosis in Cattle

The following types of ketosis are distinguished: Primary (1-8 weeks of lactation; near lactation peak), during the period of NEB; secondary (near the calving of the 1^st^ week postpartum) caused by malnutrition most often occurring as a result of appetite loss, with other conditions (e.g., indigestion or uterine and udder inflammation); and nutritional (a consequence of dietary errors). Nutritional ketosis is most common in breeding practice. Type I ketosis is most often diagnosed in 3-5 weeks of lactation. Its cause is mainly deficient energy nutrition in the postpartum period with the high demand resulting from high milk yield (peak lactation). This type of ketosis is characteristic of high-performance cows. Type II ketosis is diagnosed most often a few days after calving. This is usually due to insufficient coverage of energy demand because of insufficient DMI. The lack of appetite during this period is caused by overfeeding (fat cow syndrome). Ketosis types I and II, especially in the subclinical form, are the most common current metabolic disorders in dairy cows.

### Ketosis Symptoms

The clinical manifestations of ketosis in various stages of its development are as follows:

In Stage I of the disease, cows are more likely to eat hay and grass and avoid concentrated feed and silage. They eat sand and pebbles (pica), move reluctantly, have a shaky gait, and they hold their heads on to the litter while lying.

In Stage II of the disease, cows eat significantly less and are dull and apathetic. The milk yield decreases and animals lose weight. A high-fat content (sometimes up to 5%) and a small amount of protein are noted in the milk. A smell of acetone and fermenting fruit exist in the exhaled air. In addition, cow feces are hard, dry, and covered with mucus. Moreover, cows hold their heads low with eyes closed (dozing).

In Stage III of the disease, the cows often move forward without motives, with seizures, and abundant saliva. The animals are very sensitive to noise or touch, and they make a noise as if they were drinking (they dip their muzzle into the drinker and loudly munch). A very low-fat concentration and high βHBA amounts are noted in the blood of cows.

### Effects of Ketosis

The effects of ketosis are multifaceted. These include clinical symptoms (loss of appetite, preference of forage to concentrated feed, and acetone odor in the mouth and urine), milk composition change, production drop, a concurrent increase in diseases (*mastitis*, metritis, and displaced abomasum), and reproduction disturbance.

Several studies have described the deleterious effects of ketosis on animal health and reproduction. The effect of NEB during early lactation on later reproductive performance is well-documented, acting through the disruption of the hypothalamus–pituitary–ovary axis [35]. Other studies confirm the negative effects of SCK on reproductive efficiency [9,36,37]. CK is associated with an increase of 2-3 days to first service and a 4-10% reduction in pregnancies per artificial insemination at first service. According to Rutherford *et al*. [37], the first insemination was 4.3 times less likely to be successful in SCK cows compared with the non-SCK cows. The adjusted mean number of inseminations per pregnancy was 2.8 and 2.0 for SCK and non-SCK cows, respectively. Other researchers have identified a link between ketosis and an increased ovarian cysts incidence.

**Figure 5:**
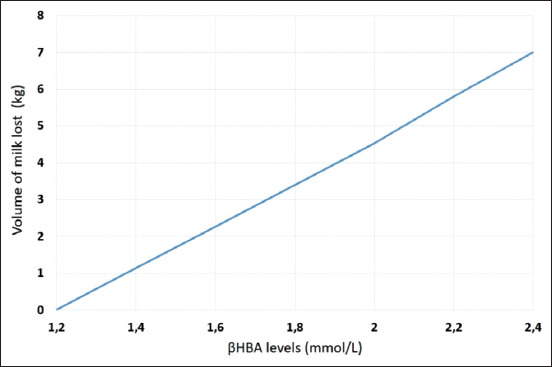
Impact of subclinical ketosis on milk production in dairy cows (own elaboration based on Oetzel [39]).

SCK leads to a milk yield decrease. It can be lower than 300 kg during one lactation [38]. SCK alone during the 1^st^ week of lactation can decrease milk yields by 2.48 kg a day, and such cows are on average 3 times more likely to be removed from the herd [10]. For every 0.1 mmol/L increase in βHBA levels, >1.2 mmol/L or 0.59 kg of milk is lost (Figure-5) [39]. Thirty-two southern Ontario Holstein herds were monitored for SCK for 2.5 years [40]. Milk ketone scores of +1 and +2 were found to be associated with a reduction in daily milk production of 1.0 and 1.4 kg of milk, respectively.

First-parity cows of the Holstein breed in Croatia were studied [22] and a significant negative effect of SCK on daily milk yield for each parity was found. A decrease in milk yield of 4.21, 2.73, 2.78, and 2.83 kg/day in each parity (i.e. parities 1, 2, 3, and 4+, respectively) was found within 35 days after the detection of SCK. The daily milk yield decreased within 35 days after the subclinical acidosis detection date with 1.4, 1.1, 2.79, and 1.74 kg day in parity class 1, 2, 3, and 4+, respectively.

High plasma concentrations of βHBA and nonesterified fatty acids (NEFA), high milk fat and citrate levels (the latter considered an early indicator), and lower milk protein and lactose concentrations are characteristics of ketosis. The levels of βHBA, NEFA, and citrate were higher in the blood of cows suffering from ketosis than in other cows [41]. The average levels of βHBA, NEFA, and citrate in plasma and milk of ketotic cows were 3.41, 1.18, and 11.45 mmol/L, respectively. They also pointed out that the amount of energy and nutrients delivered to cows after calving was not enough to cover their requirements, which caused ketosis.

The relationship between SCK incidence, determined by βHBA content in blood, and metabolic disorders in 841 Holstein cows in Croatia before and after calving was studied [24]. Two groups of cows were involved in the studies: The POS group (with clinical or SCK) and the NEG group (with no symptoms; control). Blood samples collected within 15 days after calving indicated that βHBA concentration did not have a significant effect on the whole lactation milk yield. However, cows in the POS group produced 667 kg of more milk during the lactation period than the NEG cows. Ketosis prevalence also affected fertility. The cows in the NEG group had a significantly shorter period of median days open to pregnancy compared with the POS group (124 vs. 138 days). The cows in the first lactation were less affected by SCK than the cows in the second and later lactations. More than half of the POS cows were affected by some other diseases. The relationship between the βHBA level in the plasma of blood collected in 15 days postpartum from 2290 cows in the USA and their mature-equivalent 305-day milk yield was assessed [42]. This research showed that the increase in βHBA level >10 mg/dL was accompanied by a decrease in milk yield by 393 kg in the group of multiparous cows.

The financial effects of clinical and SCK were estimated [1]. They used assumptions and input variables from a typical Dutch context. Those effects were due to lower milk production, increased cases of mastitis and other diseases, poorer reproduction performance with an increased number of inseminations, increased number of culled cows, and increased treatment costs. For at-risk cows, ketosis costs per herd and per year were €3758 which was higher than the default herd. Average herd level costs of ketosis (CK and SCK combined) were €3613 and €7371 per year for a default and a high-risk farm, respectively. The authors also found that the milk yield did not have a significant effect on the costs of ketosis.

The total SCK costs resulting from lower milk yields, discarded milk, treatment, poorer reproductive performance, and culling were tried to be estimated [43]. The total SCK costs were €130 per case per year (range, between €39 and €348). Those costs were higher for older animals. Parity three cows were nearly twice as much compared with parity one animals. The highest share of costs resulted from poorer reproductive performance (36%), and also from lower milk yields (24%) and other financial losses and expenses (20%).

## The F/P ratio in Milk as a Practical Ketosis Indicator

The fat is formed in the milk gland by attaching glycerol to fatty acids synthesized in the same gland from acetic acid (so-called *de novo* synthesis). Part of the fatty acid comes from feedstuffs or is released from adipose tissue (NEFA). As is well-known, the release of fat reserves often occurs during the perinatal period in cows of dairy breeds. Energy deficiencies in the daily ratio and excessive fatness of pregnant cows, leading to a loss of appetite, cause insufficient coverage of the cow’s energy needs. The cow becomes thinner, releasing fatty acids into the bloodstream. An important part of them enters the mammary gland and causes an increase in fat content and the share of saturated acids in milk.

The level of protein in cow’s milk depends on feedstuffs supplying energy to the microorganisms of the rumen and on the amount of protein consumed by animals. However, the microorganisms will not have anything to synthesize this component from if the amount of energy delivered to the rumen is insufficient, and the excess protein (ammonia form occurs in the rumen) will be converted by the body’s defense mechanisms into the urea and excreted (e.g., with milk).

**Figure 6:**
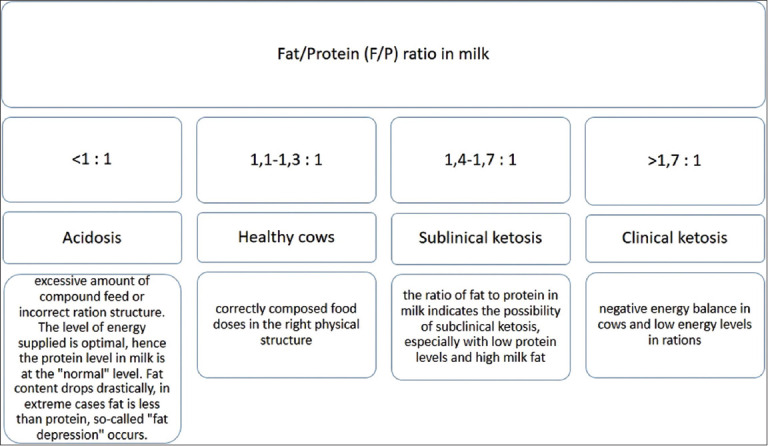
Interpretation of the rationality of cow nutrition based on the fat-protein ratio in milk.

Cattle breeders have become continuously interested in the milk F/P ratio in recent years, especially in the early lactation stages. The main reason is the possibility of its use as an important energy balance criterion in assessing cow feeding. It is assumed that normal protein content in the milk of Polish Holstein Frisian cows ranges from 3.2% to 3.6%. Normal fat content in the milk of this breed ranges between 3.5% and 4.5%, but about 4% in the case of high-performance cows [44]. The milk F/P ratio of a healthy cow fed with a well-balanced daily ratio should range from 1.1-1.3 to 1. The F/P ratio above 1.4:1 means the possibility of SCK. The clinical form of ketosis, especially with low protein content in milk and relatively high-fat content, exists when the F/P ratio exceeds 1.7:1. An F/P ratio that is too low (<1) may be indicative of subclinical acidosis, which is most commonly found when excessive feeding or abnormal physical structure of the ratio is administered. A decrease in the milk yield, deterioration of health, and, in effect, increased cow culling was noted in herds affected by acidosis (Figure-6) [44].

Particular attention should be paid to cows whose milk fat content is >5.0% in the first 41 days after calving and protein content in milk below 2.90%. If the abnormal chemical composition of milk (too much fat and not enough protein) affects more than 10% of cows that are between 7 and 60 days of lactation. Then, an analysis of the quality of cow feeding in the perinatal period is required.

The milk F/P ratio reflects the energy balance status of a cow in early lactation [45]. The primary indicator of SCK is high-fat content in milk, which is higher than the standard, in breeding practice. Another important consequence of ketosis is also an increase in the proportion of saturated acids in milk fat.

Eicher [46] recommended using the F/P ratio and the daily milk production to determine subclinical disorder (ketosis/acidosis). In addition, the F/P ratio of ≥1.5 in cows that yielded between 33 and 50 kg/day was used as an indicator of SCK, while the F/P ratio <1.0 in cows that yielded between 20 and 43 kg/day was used as an indicator of subclinical acidosis. Richardt [47] determined that a 1.5:1 value of the F/P ratio is the risk level for SCK, while Eicher [46] considered daily milk production, besides the F/P ratio, to find the indication of metabolic disorders (acidosis and ketosis). The ideal range for milk F/P ratio is 1.25:1 [48], while the upper margin is 1.33:1 [15]. According to Haas and Hofirek [49], an F/P ratio higher than 1.4:1 shows energy deficit and SCK if ketone bodies are present.

The protein content is an essential element affecting the buying-in price of milk. Milk protein price has doubled in the payment systems worldwide due to the higher suitability of protein for milk processing. Several dairies have introduced a payment system in recent years based on protein content in milk following human diet changes observed in the world when consumers prefer lower fat food. Cow breeders in most countries currently place the main emphasis on improving cow’s milk by increasing protein concentration and its efficiency and by ameliorating the F/P ratio [50].

Cows whose milk F/P ratio in breeding practice is lower are assessed as being more easily pregnant. A study showed that the F/P ratio could be a good indicator for diagnosing cows at risk of poor fertility to determine what preventive measures could be taken [51]. The F/P ratio, easily available from daily milk recording, can be used as a helpful indicator to monitor the health and fertility issues of dairy cows [52]. The authors also found that cows producing milk with a higher F/P ratio had poorer reproductive performance and higher incidence of mastitis.

### Treatment and Prevention of Ketosis

The primary method of effective prevention of type I ketosis is the constant access of cows to well-balanced daily rations with good quality components and good physical structure as well as adequate amounts of feedstuffs. In the prevention of type I ketosis, every effort should, therefore, be taken to prevent (or to seek at least a significant reduction) NEB in high-performance cows during the first lactation. This can be achieved by proper feeding of dried cows with more dry matter consumed after calving. The proper digestion of structural carbohydrates in roughage should be ensured by providing high-quality forage with the proper ratio of its dry matter to the dry matter of concentrated feed, which is a properly arranged ration should be approximately 1:1. In addition, the proper fermentation in the rumen should be ensured with an adequate amount of concentrated feed with its starch fermented to a limited extent. Such conditions are met by maize seeds present in silage or concentrated feed.

According to current knowledge, the basal diet for dried cows should be limited to 75-80% of the nutrition standards, and the amount of straw should be increased to prevent type II ketosis. This will result in a slower release of fat reserves after calving [14]. Detailed recommendations concerning ketosis prevention are keeping cows in good condition (with BCS assessment between 3.0 and 3.5 at calving time), feeding cows with the same feedstuffs 3 weeks before calving and after calving, 0.5-3 kg of concentrated feed added to the ration just before calving, careful balancing of the food ration (the proper protein-energy ratio), and avoidance of ketogenic feedstuffs (silage contain butyric acid, and feedstuffs with a high concentration of easily digestible carbohydrates).

Oral glucose precursors (e.g., feed glycerine, propylene glycol, calcium, or sodium propionate) should be administered 7-10 days before and 2 weeks after calving in herds, particularly at risk of ketosis. Their use in feed rations for cows inhibits excessive use of fat reserves, reduces the concentration of free fatty acids, and raises the levels of triglyceride and insulin. An important effect of the administration of glucose precursors is to reduce the risk of fatty liver syndrome. As a general rule, not one but a few of them should be administered because glucoplastic products increase the concentration of glucose in the blood at different rates. Daily doses are as follows: 250-300 g/day/piece approximately 2 weeks before calving and 300-350 g/day/piece after calving up to approximately 100 days of lactation, adding those products to feedstuff or a feed truck. For prophylactic purposes, a specific antiketosis drink with the following composition can be used after calving: 230 mL of propylene glycol, 450 g of calcium propionate, 50 g of rumen-protected choline, 25 g of rumen-protected methionine, 50 g of yeast, and 170 g of KCl [53]. They should be orally administered in the form of an infusion with severe symptoms of ketosis (anorexia and lack of energy). Feed glycerine is a sweet natural product made from rapeseeds. It provides the necessary energy to cows after calving and improves the taste of feedstuffs, increasing their intake and preventing ketosis. Several products – oral glucose precursors (i.e., Bolus-Enermax, Keto-Max, Dolpower Liquid, or Keto Buster 5) are available in the Polish market.

Glucose administration is also a primary medicine for the treatment of subclinical and CK. Its undoubted advantage is its concentration in the blood rises very quickly when administered intravenously. It is advisable to administer glucose together with Vitamin B_1_ or with multivitamins. Glucocorticoids are used as second-line ketosis treatment. Their advantage is the ability to raise blood glucose levels quickly and over long hours (48-72 h after administration). After glucocorticoid administration, the general health improves and the concentration of βHBA decreases. However, a temporary decrease in the daily milk yield exists at the same time [53]. It should be emphasized that this drug is not indicated in the case of type II ketosis when the complex ketosis–hepatic steatosis is well-developed.

Medication containing monensin as an active substance is also used in ketosis prevention. Monensin is a polyether antibiotic produced by *Streptomyces cinnamonensis* [54]. It reduces ketosis incidence by affecting microbial populations in the rumen when administered to cows. Monensin blocks intercellular movements of some ions and inhibits lactate-producing bacteria. Monensin treatment kills Gram-positive bacteria while Gram-negative ones are not affected. This alteration in rumen microflora triggers metabolic changes in bovine animals, improving energy, and nitrogen utilization. In effect, nutrient digestibility increases, and the risk of such digestive disorders as ruminal acidosis and ruminal bloat is reduced.

Monensin treatment lowered the risk of such disorders as ketosis, displaced abomasums, and mastitis [55]. However, it did not significantly affect the incidence of dystocia, milk fever, retained placenta, lameness, or metritis. It did not improve cattle reproductive performance either. In 2014, the effects of monensin on dairy cows in Lithuania were studied [56]. The animals were divided into groups: Groups 1 (control) and 2 (cows treated with monensin). Cows constituting Groups 1 and 2 were 30 days before calving. Cows with 1 day after calving were classified as Groups 3 (control) and 4 (treated with monensin). The βHBA concentration in the cows of Group 4 was significantly lower than in Group 3. Throughout the research, statistically significant differences exist between the βHBA concentration of control cows and the animals treated with monensin.

Kexxtone, a veterinary medication containing monensin as an active substance, is available in the Polish market together with a continuous-release intravenous bolus (bolus is given to animals orally and is passed to the rumen). Continuous release means that monensin is slowly released from the bolus. A single intraruminal bolus is given to dairy cows or heifers 3-4 weeks before the expected calving. The incidence of ketosis was 11.5% and 25.6% in the group of cows treated with Kexxtone and the placebo. The milk yield of treated cows tended to be significantly higher during the first 14 days of lactation [57].

The effect of the butyric acid content of silage on SCK incidence in pre-calving and post-calving cows was studied [58]. They investigated 20 dairy herds for a year, also determining the basic chemical composition and the content of volatile fatty acid of maize silage and grass silage. Consequently, the nutritional value of the silage varied to a large extent. It was found that 80% of 2857 urine samples gathered from cows before and after calving did not contain ketone compounds, with 16% of them from cows with elevated ketone bodies but without clinical symptoms in the other samples collected from cows that developed ketosis.

## The Need and Possibilities of Diagnosis to Manage Ketosis

Monitoring the proper nutrition of high-yielding dairy cows, especially in their perinatal period, requires reliable information on the incidence of metabolic disorders, including subclinical cases. Ketosis is the most common metabolic disease in such herds. The number of cows suffering, and, in particular, their participation in the herd, is important for daily rations, preparation for calving, feeding system, and so on.

In Poland and abroad, the following three groups of methods are used to diagnose ketosis in dairy herds:

### Determination of the level of ketone bodies in the blood

This method is primarily used by a veterinarian for the diagnosis of CK. This is an invasive and expensive method and cannot be routine. In most cases, only cows with pronounced clinical signs are subjected to blood analysis. Determining serum concentrations of NEFA, βHBA, and glucose is most useful to diagnose ketosis in cows. The determination of blood glucose does not create problems. However, a fairly serious drawback is their specificity, that is, the need to use a specialized laboratory, when determining the other two parameters.

Measuring the level of ketone bodies in the blood of dairy cows with glucose meters used in humans is possible. The meter measures electrochemical changes that occur on the test strip after blood application, which explains why it can be used in both humans and cows. The strip on which a drop of blood is released contains 3-hydroxybutyrate dehydrogenase, an enzyme oxidizing βHBA into AC. This, in turn, reduces nicotinamide adenine dinucleotide (NAD+) to NADH, and NADH is then reoxidized to NAD+. The electrical current generated during this transformation is directly proportional to the βHBA concentration in the blood. It seems very easy to measure. However, some experience is needed to take blood from cows. According to Polish law, blood can only be collected by a veterinarian. The first report using an electronic human βHBA meter (MediSense Precision, Abbott, Abingdon, UK) for dairy cows described a high correlation (r^2^ = 0.99) with βHBA concentrations determined spectrophotometrically (gold standard), and the test was considered suitable for detecting SCK in dairy cows [59].

### Determination of ketone bodies in milk or urine

Several cow side diagnostic tests (dipsticks, powders, and tablets) for ketosis are commercially available. These are designed to detect acetoacetate and, to a lesser degree, acetone, in urine (e.g., Ketostix strip, Bayer, Leverkusen, Germany) or βHBA in milk (e.g., Ketolac, Biolab, München, Germany) based on the degree of color change. The tests can be used semiquantitatively because the color change is more intense in the presence of higher levels of ketone bodies [4].

### Determination of the ketone body content (AC and βHBA) in milk during milk recording

Milk samples are taken from individual cows monthly, and animals suspected of SCK are selected on this basis. The monitoring of ketosis in Poland was introduced in the milk recording system in 2013 [60] and it is based on AC and βHBA determination in test-day (TD) milk samples by Fourier transform infrared (FTIR) spectroscopy. Moreover, the Polish Federation of Cattle Breeders and Dairy Farmers in Poland has been running this service since 2013.

### Ketosis Heritability

Using data obtained from 83,814 first lactation Holsteins cows from 1501 herds in Canada, the heritability coefficients for ketosis were estimated [61]. It was 0.02, which was in agreement with previous estimates based on linear models [61]. Higher heritability was found for βHBA_First_ (0.13) and βHBA_Max_ (0.12), whereas the βHBAS_Dit_ was much lower (0.02). Milk βHBA_First_, βHBA_Max_, and bHB_ASD_ were moderately genetically correlated with ketosis with estimates of 0.70, 0.64, and 0.82, respectively. The heritability of 0.16 for milk βHBA in dairy cows was estimated between 5 and 60 days in milk [62]. According to Lee *et al*. [63], the heritability coefficient of milk βHBA levels ranged from 0.04 to 0.17 with a mean of 0.09 for the period between 4 and 305 days in milk during three lactations. The average heritability for milk acetone concentration was 0.29, 0.29, and 0.22 for parities 1, 2, and 3, respectively.

In the Netherlands, cows have been routinely checked for ketosis in the regular milk recording scheme since 2012 [64]. The ketosis indication is based on FTIR measurements of milk AC and βHBA. Ketosis indicators also include the F/P ratio, lactation number, and month in milk. The incidence of ketosis in the Netherlands is 12.2%. Genetic parameters have been estimated with a multitrait sire model using 2.5 million ketosis observations of 1.25 million cows. Ketosis heritability for lactations 1, 2, and 3 was 0.16, 0.13, and 0.18, respectively. Genetic correlation between lactations 1 and 2, 1 and 3, and 2 and 3 was 0.81, 0.58, and 0.74, respectively. The genetic trend in the population was slightly positive, resulting in less ketosis. A correlation between ketosis and milk yield traits was slightly negative with slightly positive fertility traits (fewer ketosis results in better fertility). According to Heringstad *et a*l. [65], the posterior means of heritability of liability to ketosis in the first, second, and third lactations were 0.14, 0.16, and 0.15, respectively.

In Poland, the genetic parameters of milk F/P ratio in the first three lactations of PHF cows were calculated [66]. Data included 104,875 TD records of 6299 cows calving from 2000 to 2012. The average daily heritability of the F/P ratio ranged from 0.24 to 0.31. Moreover, the F/P ratio was negatively genetically correlated with milk yield for almost every day in milk in each lactation with means of −0.52, −0.24, and −0.05 in the first, second, and third lactations, respectively. Average genetic correlations of F/P ratio with lactose percentage and milk urea concentration were rather low or close to zero (−0.08-0.10) except for the genetic correlation with milk urea content in the second lactation (0.32).

## Conclusion

Ketosis is the most important metabolic disease in cows, being their typical production disease, in high-yielding dairy farms. This is especially true of SCK, a condition in which the animal has elevated levels of ketone bodies in the blood, milk, and urine, with often reduced glucose levels. However, clinical signs are not yet shown. Ketosis is a disease with a severe impact on animal performance and consequently on the economic well-being of dairies. Prevention usually is less costly than treatment, and the latter is associated with production losses. An essential element in ketosis prevention is keeping cows during the perinatal period in good condition. The primary method of effective prevention of type I ketosis is constant access of cows to well-balanced daily rations with good quality components and good physical structure, and adequate amounts of feedstuffs. In herds particularly at risk of ketosis, oral glucose precursors should be administered 7-10 days before and 2 weeks after calving.

## Author’s Contributions

PG: Conceived the design and conducted the literature search. The author has made a substantial, direct and intellectual contribution to the work, and approved it for publication.

## References

[1] Steeneveld W, Amuta P, Van Soest F.J.S, Jorritsma R, Hogeveen H (2020). Estimating the combined costs of clinical and subclinical ketosis in dairy cows. PLoS One.

[2] Baticz O, Tömösközi S, Vida L (2002). Concentrations of citrate and ketone bodies in cow's raw milk. Period. Polytech. Chem. Eng.

[3] Enjalbert F, Nicot M.C, Bayourthe C, Moncoulon R (2001). Ketone bodies in milk and blood of dairy cows:Relationship between concentrations and utilization for detection of subclinical ketosis. J. Dairy Sci.

[4] Guliński P (2017). Bydło Domowe Hodowla i Użytkowanie (in Polish). Wyd Nauk PWN Warszawa.

[5] Iwerson M, Falkenberg U, Voigtsberger R, Forderung D, Heuwiser W (2009). Evaluation of an electronic cowside test to detect subclinical ketosis in dairy cows. J. Dairy Sci.

[6] Kowalski M.Z, Słoniewski K, Kański J (2013). Ketoza Najważniejsza, Horoba Zawodowa Krów Mlecznych. XVI Międzynarodowa Sesja Naukowa, Zaburzenia w Rozrodzie i Produkcyjności Bydła“(in Polish). Polanica Zdrój 20-22 Czerwca.

[7] Työppönen J, Kauppinen K (1980). The stability and automatic determination of ketone bodies in blood samples taken in field conditions. Acta Vet. Scand.

[8] Djoković R, Ilić Z, Kurćubić V, Petrović M, Cincović M, Petrović M.P, Perović V.C (2019). Diagnosis of subclinical ketosis in dairy cows. Biotech. Anim. Husb.

[9] Duffield T.F, Lissemore K.D, McBride B.W, Leslie K.E (2009). Impact of hyperketonemia in early lactation dairy cows on health and production. J. Dairy Sci.

[10] McArt J.A.A, Nydam D.V, Oetzel G.R (2012). Epidemiology of subclinical ketosis in early lactation dairy cattle. J. Dairy Sci.

[11] Suthar V.S, Canelas-Raposo J, Deniz A, Heuwieser W (2013). Prevalence of subclinical ketosis and relationships with postpartum diseases in European dairy cows. J. Dairy Sci.

[12] Oetzel G.R (2007). Herd-level Ketosis Diagnosis and Risk Factors. Proceedings of the 40^th^ Annual Conference of Bovine Practitioners.

[13] Wang Y, Gao Y, Xia C, Zhang H, Qian W, Cao Y (2016). Pathway analysis of plasma different metabolites for dairy cow ketosis. Ital. J. Anim. Sci.

[14] Ranaraja U, Cho K.H, Park M.N, Choi T.J, Kim S.D, Lee J.S, Kim H.S, Do C.H (2016). Impact of environmental factors on milk β-hydroxybutyric acid and acetone levels in Holstein cattle associated with production traits. Korean J. Agric. Sci.

[15] Duffield T (2013). Wpływ Podklinicznej Ketozy na Zdrowie, Mleczność, Wyniki Rozrodu Oraz Ryzyko Brakowania Krów. XVI Międzynarodowa Sesja Naukowa, Zaburzenia w Rozrodzie i Produkcyjności Bydła“(in Polish). Polanica Zdrój 20-22 Czerwca.

[16] Brunner N, Groeger S, Raposo J.C, Bruckmaier R.M, Gross J.J (2019). Prevalence of subclinical ketosis and production diseases in dairy cows in Central and South America, Africa, Asia, Australia, New Zealand, and Eastern Europe. Transl. Anim. Sci.

[17] Buttchereit N, Stamer E, Junge W, Thaller G (2012). Genetic parameters for energy balance, fat/protein ratio, body condition score and disease traits in German Holstein cows. J. Anim. Breed. Gen.

[18] Fiorentin E.L, Zanovello S, Gato A, André L, Piovezan A.L, Alves M.V, Rocha R.X, Gonzalez F (2018). Occurrence of subclinical metabolic disorders in dairy cows from western Santa Catarina state. Brazil. Pesq. Vet. Bras.

[19] Asl A.N, Nazifi S, Ghasrodashti A.R, Olyaee A (2011). Prevalence of subclinical ketosis in dairy cattle in the southwestern Iran and detection of cutoff point for NEFA and glucose concentrations for diagnosis of subclinical ketosis. Prev. Vet. Med.

[20] Vlček M, Candrák J, Kasarda R (2016). Fat-to-protein ratio:Evaluation of metabolic disorders and milk yield. Acta Agric. Slovenica Suppl.

[21] Januś E, Borkowska D (2013). Occurrence of ketone bodies in the urine of cows during the first three months after calving and their association with milk yield. Arch. Anim. Breed.

[22] Gantner V, Bobić T, Potočnik K (2016). Prevalence of metabolic disorders and effect on subsequent daily milk quantity and quality in Holstein cows. Arch. Anim. Breed.

[23] Guliński P (2019). Prevalence of selected metabolic diseases in dairy herds in eastern Poland. Acta Sci. Pol. Zootech.

[24] Vince S, Đuričić D, Valpotić H, Gračner D, Folnožić I, Špoljarić B, Sobiech P, Samardžija M (2017). Risk factors and prevalence of subclinical ketosis in dairy cows in Croatia. Vet. Arhiv.

[25] Wildman E.E, Jones G.M, Wagner P.E, Bowman R.L (1982). A dairy cow body condition scoring system and its relationship to selected production characteristics. J. Dairy Sci.

[26] Wathes D.C, Clempson A.M, Pollot G.E (2013). Associations between lipid metabolism and fertility in the dairy cow. Reprod. Fertil. Dev.

[27] Borkowska D, Januś E, Wilgos A (2012). The effect of selected factors on changes in body condition in high-yield cows. Acta Sci. Pol. Zootech.

[28] Jankowska M, Sawa A, Gierszewski R (2012). The influence of selected factors on the condition of cows and its relationship with fertility indices [Wpływ wybranych czynników na kondycjękrów i jej związek ze wskaźnikami płodności (in Polish)]. Rocz. Nauk. PT Z.

[29] Sablik P, Januś E, Szewczuk M, Vovk S, Padzik N (2019). Effect of selected factors on the body condition of dairy cows managed in the free-stall and tie-stall housing systems. Acta Sci. Pol. Zootech.

[30] Miciński J, Pogorzelska J (2011). The effect of dairy cattle management systems on milk yield, composition and somatic cell count. Acta Sci. Pol. Zootech.

[31] Rodenburg J (1992). Body Condition Scoring of Dairy Cattle.

[32] Hemken R.W, Morris J.G, Brown W.L (1988). Nutrient Requirements of Dairy Cattle.

[33] De Vries T (2015). Predicting and Identifying Health Problems through Changes in Dairy Cow Behavior.

[34] Chalmeh A, Hajimohammadi A, Bagheri S, Jalali M (2015). Changes in serum metabolic hormone levels after glucose infusion during lactation cycles in Holstein cows. Vet. Sci. Dev.

[35] Butler W.R (2003). Energy balance relationships with follicular development, ovulation and fertility in postpartum dairy cows. Livest. Prod. Sci.

[36] Walsh R.B, Walton J.S, Kelton D.F, LeBlanc S.J, Leslie K.E, Duffield T.F (2007). The effect of subclinical ketosis in early lactation on reproductive performance of postpartum dairy cows. J. Dairy Sci.

[37] Rutherford A.J, Oikonomou G, Smith R.F (2016). The effect of subclinical ketosis on activity at estrus and reproductive performance in dairy cattle. J. Dairy Sci.

[38] Rajala-Schultz P.J, Grohn Y.T, McCulloch C.E (1999). Effects of milk fever, ketosis, and lameness on milk yield in dairy cows. J. Dairy Sci.

[39] Oetzel G.R (2012). Understanding the Impact of Subclinical Ketosis. Proceedings of the Cornell Nutrition Conference for Feed Manufacturers.

[40] Dohoo I.R, Martin S.W (1984). Subclinical ketosis:Prevalence and associations with production and disease. Can. J. Comp. Med.

[41] Yang W, Zhang B, Xu Ch, Zhang H, Xia C (2019). Effects of ketosis in dairy cows on blood biochemical parameters, milk yield and composition, and digestive capacity. J. Vet. Res.

[42] Ospina P.A, Nydam D.V, Stokol T, Overton T.R (2010). Associations of elevated nonesterified fatty acids and beta-hydroxybutyrate concentrations with early lactation reproductive performance and milk production in transition dairy cattle in the northeastern United States. J. Dairy Sci.

[43] Mostert P, Bokkers E, Van Middelaar C, Hogeveen H, De Boer I (2018). Estimating the economic impact of subclinical ketosis in dairy cattle using a dynamic stochastic simulation model. Animal.

[44] Guliński P, Salamończyk E, Młynek K (2018). Possibilities of modifying the chemical composition of cow's milk [Możliwości modyfikacji składu chemicznego mleka krów (in Polish). Wyd. Nauk. UPH w Siedlcach].

[45] Buttchereit N, Stamer E, Junge W, Thaller G (2010). Evaluation of five lactation curve models fitted for fat:Protein ratio of milk and daily energy balance. J. Dairy Sci.

[46] Eicher R (2004). Evaluation of the Metabolic and Nutritional Situation in Dairy Herds:Diagnostic Use of Milk Components.

[47] Richardt W (2004). Milk composition as an indicator of nutrition and health. Breeding.

[48] Gravert H.O (1991). Indicators for assessment of energy balance in high-yielding cows. Monat. Vet.

[49] Haas D, Hofírek B (2004). The Diagnostic Importance of Milk Components for a Human and Cows Health, CUA Prague. Proceedings of Contributions:Milk Day.

[50] Miglior F, Muir B.L, Van Doormaal B.J (2005). Selection indices in Holstein cattle of various countries. J. Dairy Sci.

[51] Löf E, Gustafsson H, Emanuelson U (2014). Factors influencing the chance of cows being pregnant 30 days after the herd voluntary waiting period. J. Dairy Sci.

[52] Negussie E, Strandén I, Mäntysaari E.A (2013). Genetic associations of test-day fat:Protein ratio with milk yield, fertility, and udder health traits in Nordic Red cattle. J. Dairy Sci.

[53] Marczuk J, Kiczorowska B, Kurek Ł, Brodzki P (2013). Advances in the diagnosis, therapy and prophylaxis of ketosis in dairy cattle [Postępy w Diagnostyce, Terapii i Profilaktyce Ketozy u Bydła Mlecznego (in Polish)]. Mag Wet, Wrzesień.

[54] Duffield T.F, Rabiee A.R, Lean I.J (2008a). A meta-analysis of the impact of monensin in lactating dairy cattle. Part 1. Metabolic effects. J. Dairy Sci.

[55] Duffield T.F, Rabiee A.R, Lean I.J (2008b). A meta-analysis of the impact of monensin in lactating dairy cattle. Part 3. health and reproduction. J. Dairy Sci.

[56] Antanaitis R, Žilaitis V, Juozaitienė V, Stoškus R, Televičius M (2015). Effects of monensin on production and energy metabolism in early lactation cows. Žemėsūkiomokslai.

[57] Losand B, Blum E, Flor J (2015). Efficacy of Kexxtone in practice. Tierärztliche Umschau.

[58] Vicente F, Rodríguez M.L, Martínez-Fernández A, Soldado A, Argamentería A, Peláez M, de la Roza-Delgado B (2014). Subclinical ketosis on dairy cows in transition period in farms with contrasting butyric acid contents in silages. Sci. World J.

[59] Jeppesen R, Enemark J.M.D, Enevoldsen C (2006). Ketone body measurement in dairy cows. Reference OS43-2 in Proceedings 24^th^ World Buiatrics Congress, Nice, France.

[60] Kowalski Z, Plyta A, Rybicka E, Jagusiak W, Słoniewski K (2015). Novel Model of Monitoring of Subclinical Ketosis in Dairy Herds in Poland Based on Monthly Milk Recording and Estimation of Ketone Bodies in Milk by FTIR Spectroscopy Technology. Vol. 19. ICAR Technical Series.

[61] Koeck A, Jamrozik J, Kistemaker G.J, Schenkel F.S, Moore R.K, Lefebvre D.M, Kelton D.F, Miglior F (2016). Genetic and phenotypic associations of milk β-hydroxybutyrate with ketosis in Canadian Holsteins. Can. J. Anim. Sci.

[62] Van der Drift S.G.A, Van Hulzen K.J.E, Teweldemedhn T.G, Jorritsma R, Nielen M, Heuven H.C.M (2012). Genetic and nongenetic variation in plasma and milk β-hydroxybutyrate and milk acetone concentrations of early-lactation dairy cows. J. Dairy Sci.

[63] Lee S, Cho K, Park M, Choi T, Kim S, Do C (2016). Genetic parameters of milk β-hydroxybutyric acid and acetone and their genetic association with milk production traits of Holstein cattle. Asian Australs. J. Anim. Sci.

[64] Vosman J.J, de Jong G, Eding H, Knijn H (2015). Genetic Evaluation for Ketosis in the Netherlands Based on FTIR Measurements. Interbull Bulletin No. 49.

[65] Heringstad B, Chang Y.M, Gianola D, Klemetsdal G (2005). Genetic analysis of clinical mastitis, milk fever, ketosis, and retained placenta in three lactations of Norwegian Red cows. J. Dairy Sci.

[66] Satoła A, Ptak E (2019). Genetic parameters of milk fat-to-protein ratio in first three lactations of Polish Holstein-Friesian cows. J. Anim. Feed Sci.

